# Loss of septal cholinergic input to lateral entorhinal cortex is an early indicator of cognitive impairment

**DOI:** 10.21203/rs.3.rs-3851086/v1

**Published:** 2024-01-11

**Authors:** Mala R. Ananth, John D. Gardus, Chuan Huang, Nikhil Palekar, Mark Slifstein, Laszlo Zaborszky, Ramin V. Parsey, David A. Talmage, Christine DeLorenzo, Lorna W. Role

**Affiliations:** National Institutes of Health; Stony Brook Medicine; Emory University School of Medicine; Stony Brook Medicine; Stony Brook Medicine; Rutgers University; Stony Brook Medicine; National Institutes of Health; Stony Brook Medicine; National Institutes of Health

**Keywords:** Cholinergic, Entorhinal Cortex, Aging, Cognition, Translational, PET, MRI

## Abstract

Although alterations to central cholinergic signaling are characteristic of late-stage cognitive deficits, the early temporal profile of these alterations and their relationship to more subtle changes in cognition are less clear. In a series of translational experiments in humans and mice, we evaluated whether changes to the cholinergic system were an early feature of pathological aging. Additional studies in mice assessed the effects of selective perturbations of cholinergic signaling on cognitive performance. We focus on the cholinergic innervation of the entorhinal cortex (EC), a region that is recognized to be susceptible early in aging and neurodegenerative disease. In human studies we recruited older adult volunteers that were physically healthy and without clinical diagnosis of cognitive impairment. Participants were divided based on their cognitive status during the intake session. Using positron emission tomography (PET) with a tracer specific for the vesicular acetylcholine transporter ([^18^F]VAT) we demonstrate that loss of cholinergic input to the EC is an early occurrence in cognitive impairment. These deficits are specific to the cholinergic circuit between the medial septum and vertical limb of the diagonal band (MS/vDB; CH1/2) to the EC. We further demonstrate impaired structural connectivity in the tracts between the MS/vDB and EC in cognitively impaired, older adults. Mouse experiments, designed to parallel the human studies, used high resolution terminal field imaging to compare normal aged mice with a genetically modified line with accumulation of amyloid beta plaques and spontaneous hyperphosphorylation of mouse tau. Across species we find that the integrity of cholinergic projections to the EC directly correlates with performance in EC-related object recognition memory tasks. We further establish the role of the MS/vDB regions of the cholinergic basal forebrain in object location memory by demonstrating that silencing EC-projecting cholinergic neurons in normal animals is sufficient to impair object recognition performance. Taken together we identify a role for acetylcholine in normal EC function and establish loss of cholinergic input to the EC as an early, conserved feature in age-related cognitive decline.

## I. Introduction

Acetylcholine is a key neuromodulator in the brain that is critical for several functions including attention, wakefulness, mood, and memory^1^. Cholinergic neurons (neurons that synthesize and release acetylcholine), coordinate neuronal activity brain-wide to promote attention to salient stimuli and facilitate learning^2^. Basal forebrain cholinergic neurons (BFCNs) span the entire rostro-caudal forebrain and send wide-spread projections to much of the brain^3^. The broad reach of these projections coupled with their functional organization grant cholinergic neurons highly flexible, context-specific control over cortical dynamics, making a strong case for the functional importance of acetylcholine in cognitive behaviors^4^.

Post-mortem analyses reveal loss of BFCNs and fragmentation of cholinergic projections in pathological aging conditions such as Alzheimer’s disease (AD)^5–7^. What these studies lack is an understanding of *when* changes to the cholinergic system occur and the importance of these changes to changes in cognition. Addressing these questions requires an early assessment of the integrity of the cholinergic system.

One of the brain regions affected earliest by age is the entorhinal cortex (EC)^8^. The EC serves as the primary input and output structure from the hippocampal formation and thus is essential for memory^9,10^. Histopathological studies reveal that accumulation of tau pathology associated with AD begins in the EC^11,12^. In addition, structural and functional alterations to the EC precede and can even predict future cognitive impairment^13–16^. Functional deficits in the entorhinal cortex, resulting in deficits in object location memory, and processing of complex objects are among the earliest reported in AD progression^17–19^. The EC receives cholinergic input from a cluster of anteriorly positioned BFCNs^1,3,20^. Whether a compromised cholinergic system influences early cognitive changes associated with EC function and integrity is not clear.

In this study we ask whether there is an association between the status of cholinergic input to the EC and cognitive ability in healthy volunteers. We then use a mouse model to study the effects of early amyloid and tau pathology on the basal forebrain cholinergic system focusing on inputs to the EC. Our findings demonstrate that in both humans and mice, compromised cholinergic input to the EC occurs early and is predictive of decreased cognitive performance, in particular, on tasks that require the EC.

## II. Results

To gain better insight into the relationship of cholinergic pathology and AD progression we asked: 1) whether alterations to the basal forebrain cholinergic system are an early feature of pathological aging in the context of EC-related functions, and 2) what is the association between an intact cholinergic system to intact cognition.

### Entorhinal cortex related functions are diminished in older adults with cognitive impairment.

**Figure 1:**
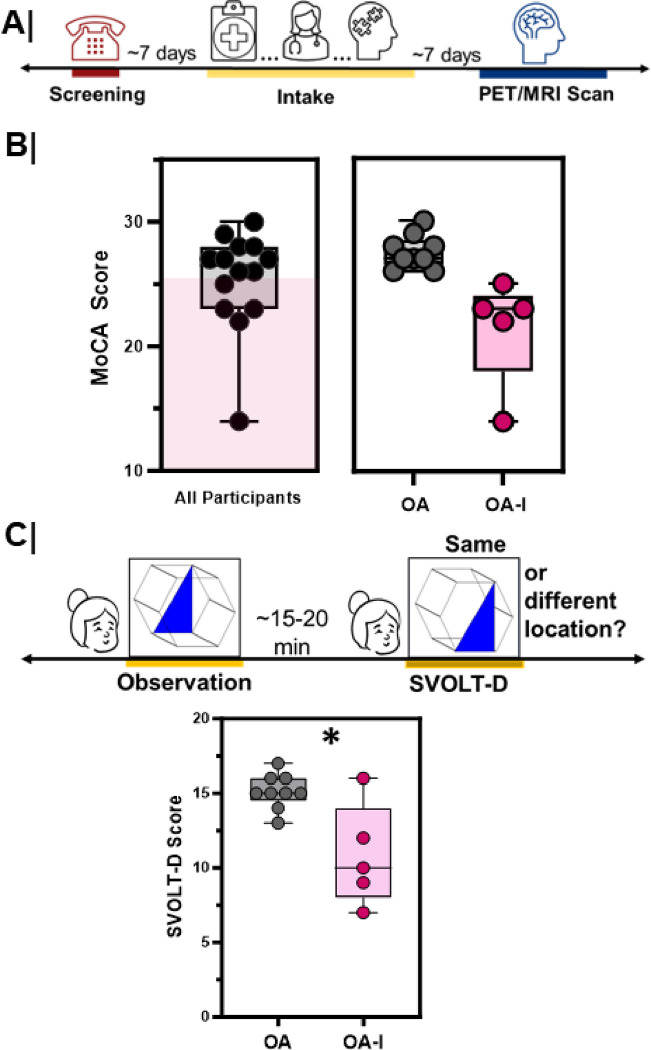
Delayed object location memory scores are lower in older adults with cognitive impairment **A.** Schematic workflow of human studies. Volunteers underwent a phone screen for initial exclusion criteria. Participants that met criteria were invited for the intake session. Each participant underwent a clinical interview, physical examination, and cognitive testing. After the intake session, participants underwent a simultaneous PET/MRI scan session. **B.** (left) Montreal Cognitive Assessment (MoCA) score across all participants was divided using the clinical threshold of 25 (top of magenta shaded box) resulting in two groups for analysis: older adults with intact cognition (**OA, Grey,** n=9) and older adults with cognitive impairment (**OA-I, Magenta,** n=5). **C.** (top) Experimental workflow highlighting the object location learning task within the Penn computerized neurocognitive battery that is administered during the intake session; (bottom) Delayed-Spatial Visual Object Learning Task (SVOLT-Delayed) between OA and OA-I participants (*, p=0.01). Older adults = **Grey**; Impaired, older adults = **Magenta.**

To answer questions about the relationship between cholinergic system integrity and AD progression, we recruited older adult volunteers (Table 1) who were active in the community, physically healthy, devoid of co-morbid mental health concerns, and had no contraindications to PET and MRI scanning procedures. None of these volunteers had clinical diagnosis of cognitive impairment (Fig. 1A).

During the intake session we evaluated the cognitive status of each participant using the Montreal Cognitive Assessment (MoCA, Fig. 1B left), a sensitive clinical assessment for mild impairments in cognition^21^. Based on their intake session MoCA score we divided our participants into two groups, older adults (OA) and impaired, older adults (OA-I) (Fig. 1B, right). All participants also underwent a standardized neurocognitive battery, the Penn Computerized Neurocognitive Battery (PennCNB), designed to assess cognitive status across multiple functional domains^22^.

The first question we asked was whether EC-related cognitive functions were altered in older adults with minor cognitive impairments. Given the importance of the EC to object location memory^19,23,24^, we used the spatial visual object learning task (SVOLT and SVOLT-delayed) within the PennCNB battery to assess EC function in our participants. SVOLT tasks test memory for complex figures. During the SVOLT observation period, participants are presented with and asked to remember a series of three-dimensional shapes with shaded sub-regions (Fig. 1C, top). Immediately after the observation period, during the SVOLT test, objects from the learning set were intermingled with new objects with different shaded regions. For each presented object, participants were asked to recall whether the object was the same or different than what was presented during the observation session. For SVOLT-delayed task (SVOLT-D) the observation and recall sessions were separated by a 15–20-minute delay during which time participants performed other cognitive tasks. Participants were re-presented with a series of objects, some new and some old, in the SVOLT-D. We quantified the number of correct responses during the immediate and delayed testing session for participants in each group. OA-I participants performed significantly worse on the SVOLT-D task than their age-matched, OA counterparts (Fig. 1C, bottom, OA vs. OA-I, p = 0.01). In contrast, performance did not significantly differ on the immediate SVOLT test (**Figure S1A**; OA vs. OA-I, p = 0.80). When we assessed the change in performance from immediate to delayed SVOLT session, we found that OA-I participants had a greater change in SVOLT performance score than OA individuals (**Figure S1B**, OA vs. OA-I, p = 0.005).

### Entorhinal cortex cholinergic density is lower in older adults with cognitive impairment and correlated with spatial memory performance.

**Figure 2:**
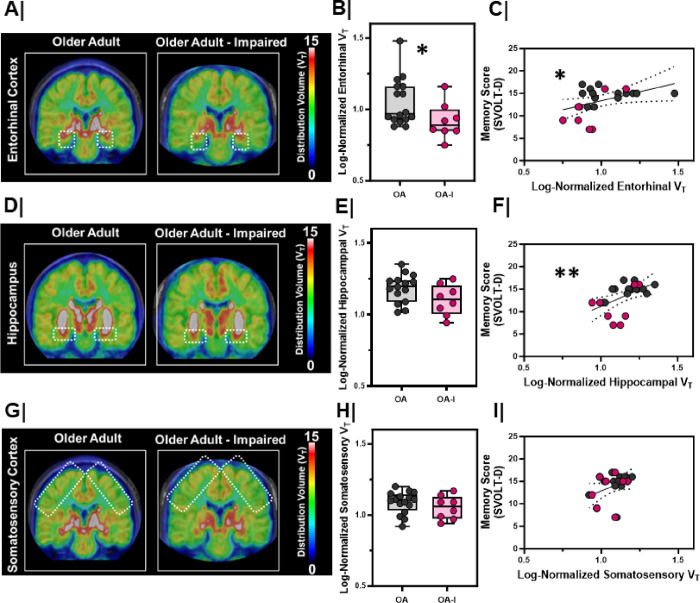
Entorhinal cortex VAT density is lower in older adults with cognitive impairment A/D/G. Averaged maps of VAChT density at each voxel from (left) older adults (n=8) vs. (right) impaired, older adults (n=4) at the level of the **A.** entorhinal cortex, **D.** hippocampal formation, **G.** somatosensory cortex. Color bar represents distribution volume (V_T_). **B/E/H.** Log-normalized right and left hemisphere V_T_ between older adults (OA, n=8) and impaired, older adults (OA-I, n=4) in the **B.** entorhinal cortex (*, p=0.04) , **E.** hippocampal formation (p=0.10), **H.** somatosensory cortex (p=0.34). Older adults = **Grey**; Impaired, older adults = **Magenta**. Hemispheres plotted individually **C/F/I.** Correlation with linear regression (black line) comparing the relationship between [18F]VAT distribution volume and delayed memory score (SVOLT-D) in the **C.** entorhinal cortex (*, r=0.47, p=0.03, linear regression significantly non-zero; confidence interval marked by dashed black lines), F. hippocampal formation (**, r=0.54, p=0.008, linear regression significantly non-zero; confidence interval marked by dashed black lines), I. somatosensory cortex (NS, r=0.13, p=0.09, linear regression not significant; confidence interval marked by dashed black lines). Older adults = **Grey**; Impaired, older adults = **Magenta**. Hemispheres plotted individually.

Next, we asked whether OA-I participants displayed differences in the synaptic integrity of cholinergic inputs in the EC. We quantified the density of the vesicular acetylcholine transporter (VAChT) *in vivo* using [^18^F]VAT Positron Emission Tomography (PET) (Fig. 2). [^18^F]VAT specifically and selectively binds to VAChT, allowing us to quantify the integrity of the cholinergic system *in vivo*^25–27^. Linear mixed models were fit with [^18^F]VAT regional distribution volume (proportional to density) as the model outcome with group (OA or OA-I), sex (M or F), and hemisphere (R or L) as fixed effects. We found a significant main effect of group (with no main effect of sex or hemisphere), where EC distribution volumes were lower in OA-I participants compared to OA participants (Fig. 2A/B, p = 0.04). In contrast, distribution volumes in other areas (fit in individual models) such as the hippocampus (Fig. 2D/E) or in the somatosensory cortex (Fig. 2G/H) did not differ between groups (hippocampus, p = 0.10; somatosensory cortex, p = 0.34).

To directly compare the relationship between cholinergic system integrity and SVOLT performance, we next fit a model with SVOLT-D performance as the outcome and EC distribution volume as a fixed effect. Larger EC distribution volume positively correlated with higher performance on the SVOLT-D task (Fig. 2C, r_S_ = 0.47, p = 0.03). We also found that hippocampal distribution volume positively correlated with SVOLT-D performance (Fig. 2F, r_S_ = 0.54, p = 0.008). In contrast, somatosensory cortex distribution volume was not significantly related to performance on the SVOLT task (Fig. 2I, r_S_ = 0.13, p = 0.09).

### MS/vDB (CH1/2) cholinergic density is lower in older adults with cognitive impairment.

**Figure 3:**
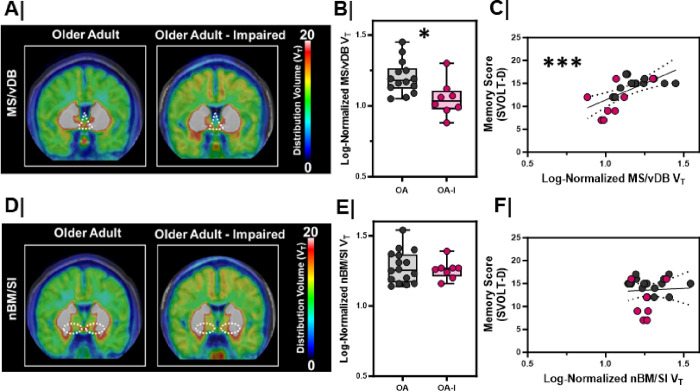
MS/vDB VAT density is lower in older adults with cognitive impairment A/D. Averaged maps of VAChT density at each voxel from (left) older adults (n=8) vs. (right) impaired, older adults (n=8) at the level of the **A.** medial septum/diagonal band (MS/vDB; CH1/CH2), **D.** nucleus basalis/substantia innominata (NBM/SI, CH4p). Color bar represents distribution volume (V_T_). **B/E.** Log-normalized right and left hemisphere V_T_ between older adults (OA, n=8) and impaired, older adults (OA-I, n=4) in the **B.** MS/vDB (*, p=0.01), **E.** NBM/SI (NS, p=0.34). Older adults = **Grey**; Impaired, older adults = **Magenta**. Hemispheres plotted individually. **C/F.** Correlation with linear regression (black line) comparing the relationship between [^18^F]VAT distribution volume and delayed memory score (SVOLT-D) in the C. MS/vDB (***, r=0.42, p<0.001, linear regression significantly non-zero; confidence interval marked by dashed black lines), **F.** NBM/SI (NS, r=0.03, p=0.80, linear regression not significant; confidence interval marked by dashed black lines). Older adults = **Grey**; Impaired, older adults = **Magenta**. Hemispheres plotted individually.

In rodents and non-human primates, cholinergic neurons in MS/vDB region innervate the EC, the hippocampal subfields and the prefrontal cortex ^3,28,29^. Using a previously validated BFCN subregion atlas^30^, we evaluated MS/vDB (CH1/2), hDB (CH3), and nBM/SI (CH4p) distribution volumes across our groups (Fig. 3/**S2**). Linear mixed models were fit with MS/vDB distribution volume as the model outcome with group (OA or OA-I), sex (M or F), and hemisphere (R or L) as fixed effects. We found that MS/vDB distribution volume was significantly lower in OA-I participants compared to OA participants (Fig. 3A/B, p = 0.01) with no effect of sex or hemisphere. This group difference was not observed in individually fit model group comparisons for nBM/SI (Fig. 3D/E, p = 0.34) or hDB (**Figure S2A**, p = 0.16) distribution volumes.

To directly compare the relationship between cholinergic integrity in BFCNs and SVOLT performance, we fit a model with SVOLT-D performance as the outcome and MS/vDB distribution volume as a fixed effect. We found larger MS/vDB distribution volumes corresponded with higher performance on the SVOLT-D task (Fig. 3C, r_S_ = 0.67, p < 0.001). In contrast, there was no relationship between SVOLT-D performance and distribution volume in the nBM (Fig. 3F, rS = 0.05, p = 0.82) or hDB (**Figure S2B**, r_S_ = 0.16, p = 0.47).

### The circuit between the MS/vDB and EC is structurally impaired in older adults with cognitive impairment.

**Figure 4:**
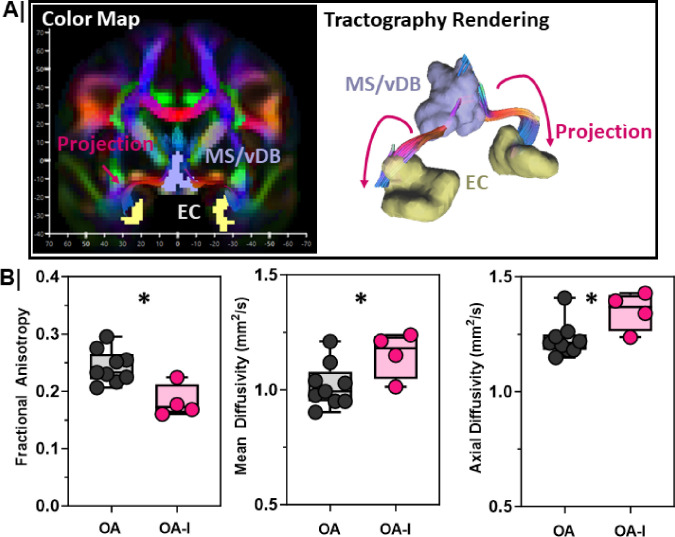
MS/vDB to entorhinal cortex connectivity is impaired in older adults with cognitive impairment **A. (Left)** Representative color map from an OA participant in coronal plane where fiber direction is represented by color. (**Right**) Corresponding 3D tractography depicts the reconstructed tract (magenta) between the MS/vDB (lavender) and EC (yellow) regions within which diffusion metrics were measured between older adults (OA, n=9) and impaired, older adults (OA-I, n=4). **B.** Diffusion metrics along the reconstructed tracts were compared between older adult (OA) and impaired, older adults (OA-I). Metrics include: fractional anisotropy (left; *, p=0.01), mean diffusivity (middle; *, p=0.05), and axial diffusivity (right; *, p=0.05) between OA and OA-I participants. Older adults = **Grey**; Impaired, older adults = **Magenta**.

We found that OA-I participants had lower distribution volumes in the MS/vDB (Fig. 3), and the EC (Fig. 2), that correlates with poor SVOLT-D performance. Next, we asked whether there were deficits in the structural connectivity between the MS/vDB and EC in OA-I participants compared to their OA counterparts. Using diffusion weighted MRI images acquired simultaneously to the PET imaging, we reconstructed tracts between the MS/vDB (set as seed region) and the EC (ROI/End region) (Fig. 4A). We quantified diffusion metrics along the rendered tract (Fig. 4A, right) between the MS/vDB and the EC in each participant. We found reductions in fractional anisotropy (FA, Fig. 4B-left, p = 0.01), and elevated mean diffusivity (MD, Fig. 4B-middle, p = 0.05), and axial diffusivity (AD, Fig. 4B-right, p = 0.05) in OA-I participants compared to OA counterparts. These data are consistent with disordered, structurally impaired tracts between the MS/vDB and EC.

### The displaced object recognition (DOR) behavioral task activates the lateral entorhinal cortex.

The data presented above establishes a relationship between EC related memory performance and the integrity of the MS/vDB cholinergic projection to the EC. To gain insight into the function of acetylcholine in the EC, we turned to animal models. We focused our questions on the cholinergic circuit to the lateral entorhinal cortex (LEC) given the LECs early role in age-related vulnerabilities ^12,14,16–19^.

**Figure 5:**
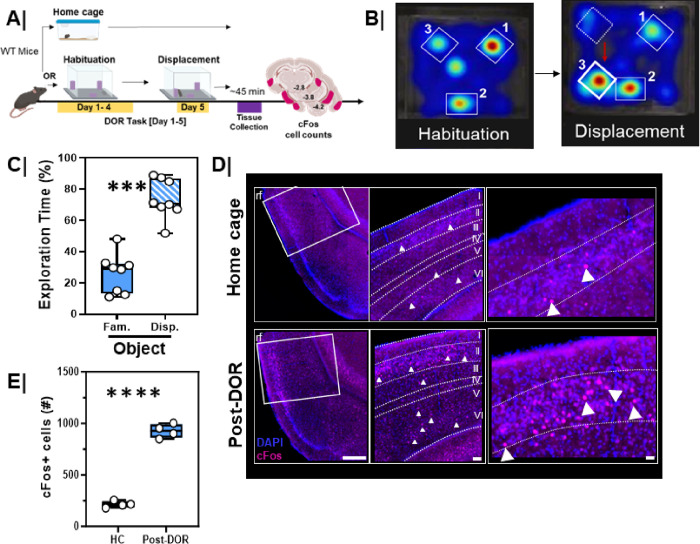
The displaced object recognition (DOR) behavioral task engages the lateral entorhinal cortex in rodents **A.** (left, top) Experimental workflow. WT, male and female mice, were either maintained in the home cage or underwent the Displaced Object Recognition (DOR) task including habituation sessions (HAB, days 1–4) and then a displacement session (day 5). Brain tissue was harvested 45 min after the behavior session and processed for cFos immunoreactivity. **B.** Representative heat maps showing arena exploration during the habituation and displacement sessions. Red arrow denotes direction of displacement. Hot colors on the heatmap represent more time spent exploring while cool colors represent less time spent exploring. **C.** Quantification of percent object exploration in male and female mice between the two identically shaped familiar objects after one of the objects was displaced (n=8/group; Fam vs. Disp, ***, p=0.0002). **D.** (left) Low-magnification (scale 200um), (middle) mid-magnification (scale 50um), and high-magnification (scale 20 um) inset of cFos immunoreactivity in (top) home cage (HC) and (bottom) following displacement session of the DOR task. **E.** Quantification of total cFos activation in the LEC following the displacement (n=4) session of the DOR task compared to home cage only (n=4) (****, p=0.0001).

In rodents, the displaced object recognition (DOR) task closely mirrors the object location task administered in human studies. We first evaluated the potential of the DOR task to engage the mouse LEC. Mice were habituated to an environment with objects in set locations over four consecutive days (Fig. 5A, Habituation). On the test day, Day 5, one object was displaced to a new location within the arena (Fig. 5A, Displacement). Typically, mice explore the newly displaced object more than they would the non-displaced objects, as illustrated in the sample heatmaps of Habituation vs Displacement (Fig. 5B) where object 3 was moved to a new location. We quantified the total time spent exploring all objects during both the habituation and displacement sessions in males and females. Because males and females displayed identical behaviors, all of our subsequent analyses collapsed sex as a variable and included both male and female mice for all experiments. Total exploration did not differ between habituation and displacement sessions, so subsequent results are expressed as percent exploration time. During the displacement session, three-month-old WT animals spent more time exploring the displaced object compared to the familiar object (Fig. 5C, p = 0.0002).

To test for activation of neurons in the LEC we assessed immunoreactivity for the expression of the immediate early gene product cFos 45 mins following the DOR task (Fig. 5D/**S3A**). Significantly more LEC neurons expressed cFos following the DOR session when compared to mice maintained in their home cages (HC; Fig. 5D/E, p = 0.0001). We also evaluated cFos expression in mice following habituation for five days (HAB), following a single session of object exploration (Novel Obj.), and following exploration of an empty arena for five days (OF). Consistent with the role of the EC in object encoding 19,23,24 we found elevated cFos immunoreactivity in the habituation and novel object groups compared to the open field and home cage control conditions (**Figure S3B**, HC/OF vs. Novel Obj/HAB, p < 0.01). DOR also significantly elevated cFos in the LEC compared to novel object or habituation conditions (**Figure S3B**, Novel Obj/HAB vs. DOR, p < 0.005) and open field or home cage conditions (**Figure S3B**, HC/OF vs. DOR, p < 0.0001).

### Aß ^+^ Tau ^+^ mice have accelerated pathology in lateral entorhinal cortex.

**Figure 6:**
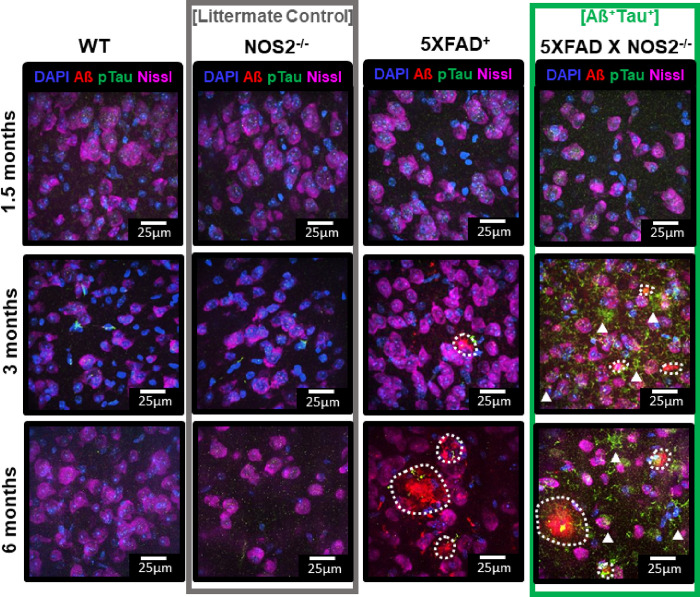
5XFAD X NOS2^−/−^ Animals have Aß and PHF-Tau Accumulation in Lateral Entorhinal Cortex by 3 months Representative images of Aß (red) and pTau (green) immunostaining in WT, NOS2^−/−^ , 5XFAD + , and NOS2^−/−^ X 5XFAD^+^ mice across (first row) 1.5-month, (second row) 3-month, (third row) 6-month mice from lateral entorhinal cortex with DAPI (nuclear stain, blue) and Nissl (neuronal stain, magenta) for reference. At 3 months of age and onwards both Aß and phospho-tau pathology are present in NOS2^−/−^ X 5XFAD^+^ animals. Approximate outlines of Aß plaques shown by white dotted line; phosphoTau accumulation is denoted with white arrows. NOS2^−/−^ X 5XFAD^+^ are herein referred to as “**Aß**^**+**^**Tau**^**+**^” animals. NOS2^−/−^ mice are herein referred to as “**Controls**.” **Green vertical box** denotes images from **Aß**^**+**^**Tau**^**+**^ animals. **Gray vertical box** denotes images from littermate controls.

The DOR task provides a quantifiable measure of behavioral performance and engagement of the LEC^23,24^. To answer questions about the relationship between cholinergic system integrity and LEC function in AD progression, we utilized a mouse model where deletion of NOS2 in Aß overexpressing mice (5XFAD) resulted in generation of hyperphosphorylated mouse tau (5XFAD X NOS2^−/−^, Fig. 6). It was previously found that deletion of NOS2 in APP-overexpressing mice (APPSwDI, 3xFAD) led to elevated accumulation of amyloid beta plaques and spontaneous generation of hyperphosphorylated mouse tau (PHF-tau) ^31–33^. We compared Aß and PHF-tau accumulation in the LEC in the 5XFAD X NOS2^−/−^ mice with genetic controls (C57, NOS2^−/−^, and 5XFAD) at 1.5, 3, and 6 months of age (Fig. 6). Aß plaques accumulated in 5XFAD + and 5XFAD X NOS2^−/−^ animals (Fig. 6, Aß red plaques in third and fourth columns). No Aß accumulation was seen in WT or NOS2^−/−^ mice (Fig. 6, first and second columns). Hyperphosphorylated-tau was only detected in 5XFAD X NOS2^−/−^, appearing by 3-months of age (Fig. 6, pTau, green aggregates in fourth column vs. rest). For subsequent experiments, 3-month 5XFAD X NOS2^−/−^ (Aß^+^Tau^+^), were compared to NOS2^−/−^ littermate controls (e.g., Fig. 6, littermate controls – second column vs. Aß^+^Tau^+^ mice – fourth column).

### Aß ^+^ Tau ^+^ mice have impaired DOR performance and impaired activation of the lateral entorhinal cortex.

**Figure 7:**
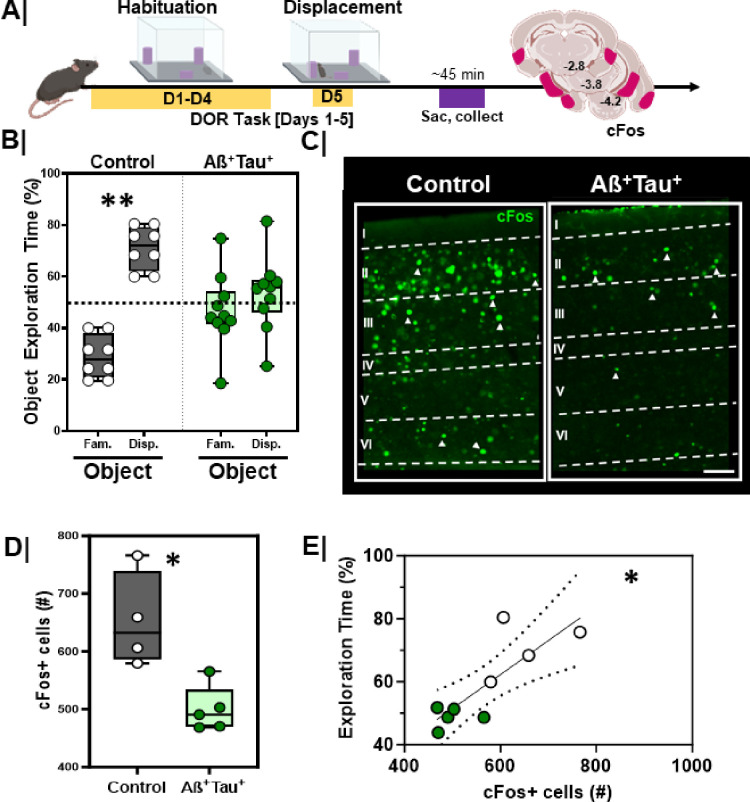
LEC activation and DOR performance is lower in Aß^+^Tau^+^ mice **A.** Experimental workflow. Aß^+^Tau^+^ animals were assessed in the displaced object recognition (DOR) task followed by tissue harvesting and cFos immunostaining; **B.** Quantification of percent time spent exploring familiar vs. displaced object during the displacement session between control (n=8; **, p=0.002) and Aß^+^Tau^+^ animals (n=10; p=0.22). Controls = **Grey box**; Aß^+^Tau^+^ = **Green box**. **C.** Representative high magnification images of LEC cFos activation during the displacement session in control (left) vs. Aß^+^Tau^+^ (right) animals; scale 50um **D.** Quantification of total cFos activation in the EC during the displacement test in control (n=4) vs Aß^+^Tau^+^ (n=5) animals (*, p=0.02). Controls = **Grey box**; Aß^+^Tau^+^ = **Green box. E.** Correlation plot with linear regression comparing the relationship between EC cFos activation (cFos+) and DOR performance in control vs Aß^+^Tau^+^ animals (*, r=0.73, p=0.03, linear regression is significantly non-zero); Control animals = white dots. Aß +Tau + animals = **Green dots**.

We quantified performance of Aß^+^Tau^+^ mice and littermate controls in the DOR task (Fig. 7). Exploration of objects was equivalent between groups during the habituation session (**Figure S4A/B**; Control, p = 0.69; Aß^+^Tau^+^, p > 0.99). During the displacement session, littermate controls spent more time exploring the displaced object (Fig. 7B; Grey bars, p = 0.002), whereas Aß^+^Tau^+^ animals spent about equal time in the exploration of both displaced and familiar objects (Fig. 7B; Green bars, p = 0.22).

To test whether this altered performance was related to impaired activation of the LEC, we assessed cFos immunoreactivity following the DOR task. Mice were sacrificed 45 min following the test session (Fig. 7A). Aß^+^Tau^+^ animals had fewer activated neurons in the LEC following DOR than littermate controls (Fig. 7C/D, p = 0.02), with overall performance in DOR directly correlating with cFos + cells in the LEC (Fig. 7E, rS=0.73, p = 0.03).

### MS/vDB cholinergic neurons project to the lateral entorhinal cortex and are activated by the DOR behavioral task.

**Figure 8:**
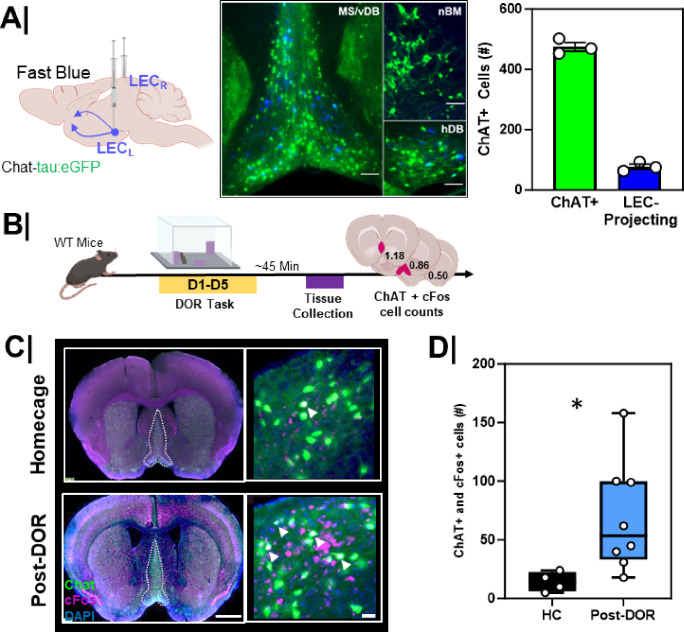
MS/vDB cholinergic neurons that project to LEC are activated by DOR **A.** (left) Schematic showing experimental workflow. Chat-tau:eGFP mice were injected with a retrograde tracer (Fast Blue) into EC followed by tissue harvesting; (middle) Representative low magnification image of LEC-projecting MS/DBB cholinergic neurons; note the predominance of blue LEC backlabeling in the medial septum/vertical diagonal band (MS/vDB) as opposed to the nucleus basalis (nBM) or horizontal diagonal band (hDB). (right) Quantification of LEC projecting cholinergic neurons in MS/vDB region of the basal forebrain as compared to total cholinergic neurons (n=3). **B.** Experimental workflow. WT mice underwent displaced object recognition (DOR) task followed by tissue harvesting and cFos immunostaining; **C.** (left) Low magnification (scale bar = 1mm) and (right) high magnification (scale bar = 50um) inset of MS/vDB cFos activation during the (top) home cage & (bottom) following the displacement session; White outline in low magnification images denote the MS/vDB region. White arrows denote cFos+ and ChAT+ (activated cholinergic) neurons within the MS/vDB. **D.** Quantification of total cFos activation in the MS/vDB following the displacement (n=8) session of the DOR task compared to home cage only (n=4) (*, p=0.01).

We next investigated the cholinergic input to the LEC in Aß^+^Tau^+^ mice. To identify the LEC-projecting subpopulation of cholinergic neurons, we injected with a retrograde tracer, Fast Blue, into the LEC of 3-month-old ChAT-tau:eGFP mice^34^ (Fig. 8A, left). In this line, all cholinergic neurons and processes are labeled with a green fluorescent protein (GFP). We found back-labeled cholinergic neurons (Fast Blue + and ChAT+) primarily in the MS and vDB, with the remainder in the hDB. No back-labeled cells were found in the nBM. Back-labeled cells represented about 10% of MS/vDB cholinergic neurons (Fig. 8A, right). To determine whether MS/vDB cholinergic neurons were activated during the DOR task, we evaluated cFos immunoreactivity in the MS/vDB of 3-month-old wild-type mice after DOR behavioral testing (Fig. 8B). Mice were sacrificed 45 min after behavior and were compared to animals that never left their home cage (Fig. 8C). We found the total number of cFos + cholinergic neurons in the MS/vDB following the displacement test (Fig. 8C/D) was significantly greater compared to home cage controls (p = 0.01).

### Entorhinal Cortex Terminal Field Density is Lower in Aß+Tau + mice.

**Figure 9:**
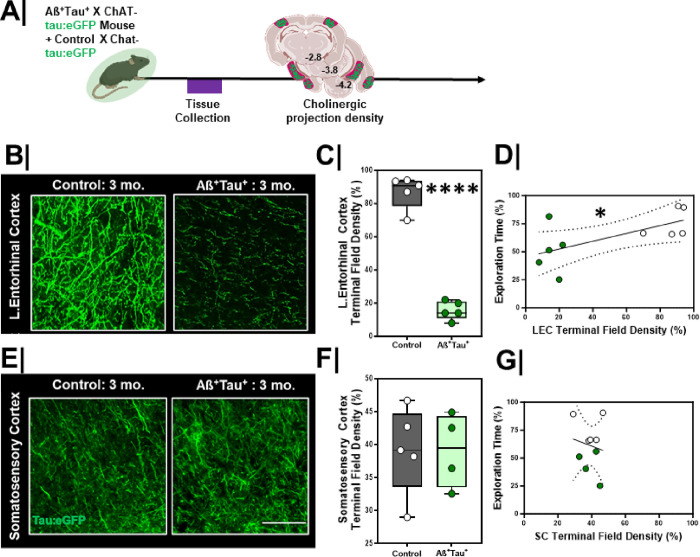
Lateral entorhinal cortex cholinergic terminal density is lower in Aß^+^Tau^+^ mice **A.** Experimental workflow. Aß^+^Tau^+^ animals were crossed to ChAT-tau:eGFP mice for evaluation of cholinergic terminal field integrity. **B/E.** High magnification representative confocal images of **A.** entorhinal cortex (EC) and **D.** somatosensory cortex (SC, bottom) in control (left) vs. Aß^+^Tau^+^ animals (right). Scale bar = 50um. **C/F.** Quantification of cholinergic terminal field density in **B.** entorhinal cortex (top, n=5/group; ****, p<0.0001 and **E.** somatosensory cortex (bottom, control n=5 + Aß^+^Tau^+^ n=4; p=0.90) between control and Aß^+^Tau^+^ animals. Controls = **Grey box**; Aß^+^Tau^+^ = **Green box**. **D/G.** Correlation plots with linear regression line comparing the relationship between cholinergic terminal field density and DOR performance in the **C.** EC (left; *, r=0.64, p=0.05, linear regression significantly non-zero; confidence intervals denoted with dashed black lines) and **F.** SC (right; r=0.15, p=0.69, linear regression not significant; confidence intervals denoted with dashed black lines). Controls = White dots; Aß^+^Tau^+^ = **Green dots**.

Given the deficits in EC-related DOR performance, next we asked whether there were changes in the synaptic integrity of cholinergic inputs in the LEC in Aß^+^Tau^+^ mice. We crossed Aß^+^Tau^+^ to Chat-tau:eGFP mice (Fig. 9A/9B) and evaluated cholinergic terminal field density in the LEC. We found that Aß^+^Tau^+^ animals had significantly lower cholinergic terminal field density in the LEC at 3-months compared to littermate controls (Fig. 9B/9C, p < 0.0001). In contrast, cholinergic terminal field density in other cortical areas such as the somatosensory cortex did not significantly differ between groups (Fig. 9E/9F p = 0.90). We found that cholinergic terminal field density in Aß^+^Tau^+^ mice did not differ from littermate controls at 1.5 months (**Figure S5A/C, left column**, p = 0.55), but did differ by 3 months (Fig. 9B/9C and **Figure S5A/C, right column**, p < 0.0001). As the ChAT-tau:eGFP offers a complimentary set of information to VAChT density, we additionally compared VAChT immunoreactivity in the LEC between Aß^+^Tau^+^ and littermate controls (**Figure S5B/S5D**), offering a more direct comparison to [^18^F]VAT distribution volumes in humans. We found that VAChT density was significantly lower in 3-month-old Aß^+^Tau^+^ mice as compared to littermate controls (**Figure S5B/S5D** p = 0.03). These results parallel our findings with the tau:eGFP experiments and our observations using [^18^F]VAT PET in OA-I vs. OA participants.

We next compared the relationship between cholinergic system integrity and DOR performance. We found that greater cholinergic input in the EC correlated with better performance on the DOR task (Fig. 9D, r_S_ = 0.64, p = 0.05). No correlation was found between somatosensory cortex cholinergic terminal field density and DOR performance (Fig. 9G, r_S_ = 0.15, p = 0.69).

### Baseline Entorhinal Cortex Activity is Elevated in Aß+Tau + mice.

We found that Aß+Tau + mice had poor performance on the DOR task and displayed blunted activation (as measured by cFos immunoreactivity) in the LEC following DOR. We also found that Aß+Tau + mice have lower cholinergic input in the LEC. To test whether these changes were accompanied by changes in baseline firing rates, we evaluated the activity in LEC in Aß+Tau + mice compared to littermate controls using in vivo anesthetized recording (**Figure S5E**). Electrodes were placed in the LEC in control and Aß^+^Tau^+^ mice and single unit baseline activity was recorded. We found Aß+Tau + mice displayed an overall elevated firing rate, with a disorganized firing pattern, at baseline compared to control animals (**Figure S5F/G**, p = 0.012).

### MS/vDB cholinergic neurons are functionally impaired in Aß ^+^ Tau ^+^ mice.

**Figure 10:**
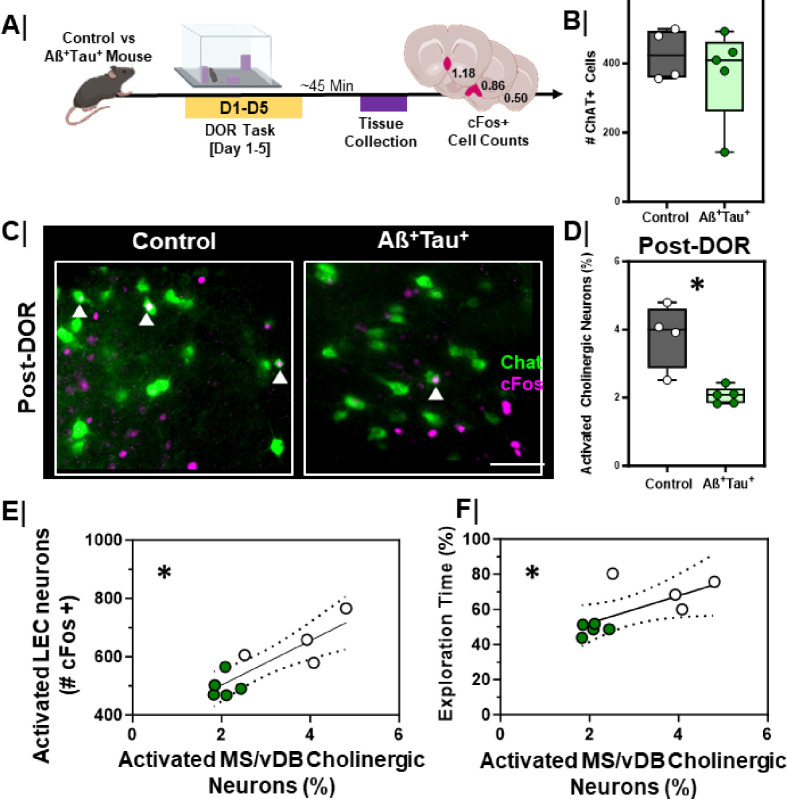
Activation of MS/vDB cholinergic neurons is lower in Aß^+^Tau^+^ animals and correlates with LEC activation and DOR performance **A.** Experimental workflow. Aß^+^Tau^+^ animals are put through the displaced object recognition (DOR) task followed by tissue harvesting and cFos immunostaining; **B.** Quantification of number of ChAT+ neurons in MS/vDB of control (n=4) vs. Aß +Tau + (n=5) animals. Controls = **Grey box**; Aß^+^Tau^+^ = **Green box. C.** Representative high magnification images of MS/DB cFos activation following the displacement session of the DOR task in control (left) vs. Aß^+^Tau^+^ (right) animals; scale bar = 50um. White arrows denote cFos+ and ChAT+ (activated cholinergic) neurons. **D.** Quantification of % activated cholinergic neurons in the MS/DB of control (n=4) vs Aß^+^Tau^+^ (n=5) animals (*, p=0.02) following the displacement session of the DOR task. Controls = **Grey box**; Aß^+^Tau^+^ = **Green box. E.** Correlation plot with linear regression line comparing the relationship between percent activated cholinergic neurons (ChAT+ and cFos+) in the MS/DB and LEC cFos activation (cFos+) following the displacement test in control (n=5) and Aß^+^Tau^+^ (n=5) animals (*, r=0.77, p=0.02, linear regression significantly non-zero). Controls = White dots; Aß^+^Tau^+^ = **Green dots. F.** Correlation plot with linear regression comparing the relationship between percent activated cholinergic neurons (ChAT+ and cFos+) in the MS/DB following DOR displacement session and DOR performance in control (n=4) vs Aß^+^Tau^+^ (n=5) animals (*, r=0.80, p=0.01, linear regression is significantly non-zero); Controls = White dots; Aß^+^Tau^+^ = **Green dots**.

Aß^+^Tau^+^ mice display impaired DOR performance, lower cholinergic input in the EC, and impaired engagement of the EC following DOR. To test whether these changes corresponded with functional changes to MS/vDB cholinergic neurons following DOR performance, we evaluated the expression of cFos in MS/vDB cholinergic neurons in the Aß^+^Tau^+^ mice after DOR performance (Fig. 10A). Mice were sacrificed 45 min after the test session to assess cFos immunoreactivity in the MS/vDB (Fig. 10A). Although there was no difference in the total number of ChAT + neurons in control vs. Aß^+^Tau^+^ mice (Fig. 10B, p = 0.90), Aß^+^Tau^+^ mice had fewer activated cholinergic neurons following DOR than littermate controls (Fig. 10C/D, p = 0.02).

We next compared the relationship between cFos activation of the MS/vDB and cFos activation of the LEC following DOR (Fig. 10E). We found a significant positive correlation between MS/vDB cholinergic neuron activation and EC activation, where more cFos + cholinergic neurons correlated with more cFos + cells in the LEC (Fig. 10E, r_S_ = 0.77, p = 0.02). We also examined the relationship between cFos activation of the MS/vDB and DOR performance (Fig. 10F) and found a significant positive correlation between cFos + cholinergic neurons in the MS/vDB of mice and performance on the DOR task (Fig. 10F, rS = 0.80, p = 0.01).

### Lateral entorhinal cortex projecting cholinergic neurons are necessary for proper DOR performance.

**Figure 11:**
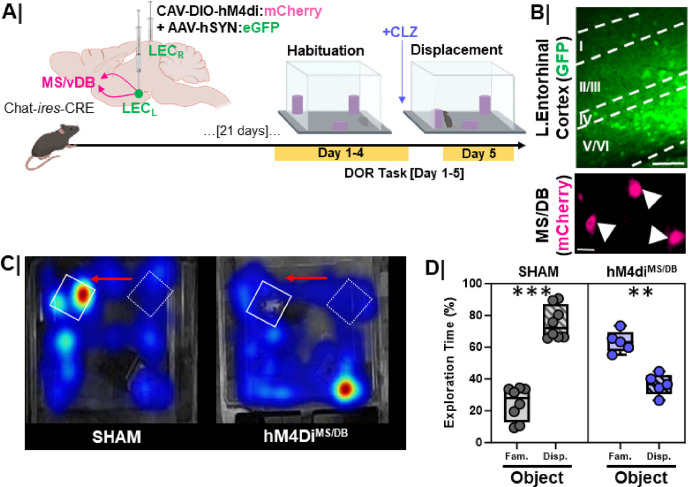
Silencing lateral entorhinal cortex projecting cholinergic neurons in young WT mice impairs DOR performance **A.** Experimental workflow. Young, 3-month-old, chat-cre animals were injected with a retrograde inhibitory DREADD construct (CAV-DIO-hM4di:**mCherry**) and a GFP control virus (AAV-hSYN:**eGFP**) to mark the injection site in LEC for cell-type specific and projection specific, manipulation of cholinergic signaling (to the EC) during the displaced object recognition task (DOR). Clozapine (0.1 mg/kg) was administered via i.p. injection (to activate the DREADD) 10 minutes prior to the displacement session (day 5); **B.** (Top) Representative image of LEC injection site, scale bar = 50um. (Bottom) Representative image of hM4Di-expressing neurons in the MS/DB, demonstrating successful, Cre-specific back-labeling of EC-projecting cholinergic neurons, scale bar = 25um; **C.** Representative heatmap showing arena exploration in control sham (left) vs. hM4di^MS/DB^ inhibition group; Red arrows denote direction of displacement. Hot colors on the heatmap represent more time spent exploring while cool colors represent less time spent exploring. **D.** Quantification of percent object exploration between the objects in control (n=7; ***, p=0.0002) vs. hM4di^MS/DB^ (n=5; **, p=0.008) mice. Data indicate the preference for the displaced object is blocked by inhibition of cholinergic projection neurons residing in the MS/vDB. Controls = **Grey box**; hM4di^MS/DB^ = **Purple box**.

Activation of cholinergic neurons correlated with both EC activation during DOR, and normal DOR performance. To determine whether cholinergic input to LEC is required for normal DOR performance, we injected the LEC of Chat-IRES-Cre-Δneo mice with CAV_2_-DIO-hM4Di.mCherry and AAV_9_-hSyn-eGFP (DREADDi animals) or AAV_9_-hSyn-eGFP alone (control animals) (Fig. 11A). DREADDi and control animals were administered clozapine (CLZ, i.p.) 10 minutes prior to the displacement test session (Fig. 11A). Tissue was processed for relocalization of the injection site and verification of mCherry + cells in the MS/vDB (Fig. 11B). Clozapine injection had no significant effect on control animal behavior; animals spent more time exploring the displaced object than the familiar object (Fig. 11C/D **grey bars**, p = 0.0002). Inhibiting MS/vDB cholinergic neurons resulted in less time exploring the displaced object compared to the familiar object (Fig. 11C/D **purple bars**, p = 0.008). Thus, activity of entorhinal cortex-projecting cholinergic neurons was required for proper DOR performance.

## III. Discussion

In this detailed PET/MRI study of 14 aging humans and parallel anatomical and functional experiments on over 100 WT and genetically modified mice, we find that cholinergic input from the MS/vDB to the EC begins to deteriorate at early stages of cognitive impairment. In both species, the decrease in cholinergic terminal field integrity in the EC correlates with impaired performance on EC related on object location memory tasks. Furthermore, selective manipulations of the cholinergic system in mice impairs object location memory, mimicking age-related disturbances to EC function. These data are consistent with a primary role of acetylcholine in the cognitive deficits associated with early EC dysfunction in aging.

### Loss of Cholinergic Input in the [Lateral] Entorhinal Cortex is an Early Feature of Cognitive Decline

Prior post-mortem studies report loss of cholinergic markers and neurons in individuals with Alzheimer’s disease^5–7^. Using recent advances in in vivo imaging methodology^25–27^, we evaluated cholinergic synaptic integrity in the human EC, a region that is uniquely susceptible to aging and neurodegenerative disease. We find that loss of cholinergic input to the EC is, in fact, an early occurrence in the progression of cognitive decline in both humans and mice, revealing a specific vulnerability in the circuit between the MS/vDB (CH1/2) of the basal forebrain and the EC. Furthermore, we find a strong relationship between the integrity of cholinergic input in the EC and hippocampus and performance on object location memory tasks.

EC function is required for object location memory in primates and in rodents^17,19,24,35–37^. Likewise, projection patterns of the basal forebrain cholinergic system are conserved across species^3,38,39^. Here we find that in addition to the anatomical connectivity, the function of the EC-projecting cholinergic circuit, and the age-related vulnerabilities associated with this circuit are also conserved in humans and mice. As such, cross-species investigation of the underlying mechanisms of age-related changes in cognition are of clinical relevance.

### Functional Changes in the Basal Forebrain Projection to the Entorhinal Cortex Precede Cell Loss

We evaluated the integrity of basal forebrain cholinergic nuclei in our human cohort using an established segmentation atlas^30^. The distribution of [^18^F]VAT accurately reports VAChT expression in the brain, and can be used to measure cholinergic projections as well as cholinergic cell bodies^40^. We find that early in the progression of cognitive impairment, MS/vDB (CH1/2) cholinergic integrity (volume of distribution) is lower in OA-I participants. Though our data are cross-sectional, this likely reflects changes in VAChT levels in cholinergic soma and/or local axons. We did not find decreased cholinergic integrity in hDB (CH3) or nBM/SI (CH4) nuclei.

Leveraging higher resolution imaging capability in mice, we found that counts of MS/vDB cholinergic neurons as assessed by ChAT immunoreactivity did not differ between control and cognitively impaired Aß^+^Tau^+^ mice. Although the numbers of MS/vDB cholinergic neurons did not differ, the engagement of MS/vDB cholinergic neurons by object location memory was significantly lower in the cognitively impaired Aß^+^Tau^+^ mice. If conserved across species, this suggests that lower EC volume of distribution found in OA-I participants reflects decreased VAChT expression, and not a loss of cholinergic neuron number per se.

A prior seed-to-searchlight MRI analysis found that basal forebrain nuclear degeneration covaries with cortical degeneration, reflective of their projections^41^. Our data are consistent with the hypothesis that the local EC cholinergic terminal integrity and the integrity of EC-projecting cholinergic circuits are amongst the earliest affected. A targeted diffusion imaging analysis of the structural integrity of the circuit between the MS/vDB and the EC in humans was consistent with disordered, fragmented, and structurally impaired connectivity. Affected parameters included decreases in the anisotropic diffusion and increases in mean and axial diffusivity, consistent with a loss of white matter integrity as reported in neurodegenerative conditions^42^ and advanced age^43^.

### Acetylcholine Plays a Critical role in Entorhinal Cortex Function and Performance

The EC receives a dense projection from the medial septum and diagonal band nucleus consisting of glutamatergic, GABAergic, and cholinergic input^44^. Cholinergic terminals in the LEC synapse onto both principal neurons and interneurons that express a variety of muscarinic and nicotinic acetylcholine receptors^45^. As such, the net activity of acetylcholine in the LEC is complex and is likely to be coordinated in a behaviorally relevant manner. In our mouse studies, we find that the degree of activation of the LEC and the cholinergic MS/DB as measured by cFos immunoreactivity strongly relates to performance on the object location memory task (DOR), and that there are significant correlations between the extent of MS/vDB cholinergic activation, the integrity of their projections to the EC, and the activation of EC neurons and DOR performance. Perhaps most compelling, chemogenetic inhibition of EC projecting cholinergic neurons in healthy young mice disrupts DOR performance. Our results are consistent with the hypothesis that appropriate ACh tone in the EC is important for proper EC function.

Aß^+^Tau^+^ animals display elevated baseline activity compared to littermate controls. This finding of hyperexcitability in aging circuits is congruent with a growing body of literature^14,46,47^. There are several mechanisms that could underlie this phenotype including Aß accumulation contributing to downstream synaptic dysfunction^48^, imbalance of the excitation:inhibition balance^48^ potentially due to loss of cholinergic input, and an overall decrease in GABAergic tone^46^. We propose that loss of EC ACh tone (loss of cholinergic input) results in elevation of circuit activity (increased baseline excitability), and an inability to further activate the EC in a behaviorally relevant manner (impaired cFos activation of MS/DB and EC), resulting in impaired object location memory performance. In support of this, silencing EC-projecting cholinergic input in normal animals is sufficient to dramatically affect object location memory performance.

### Quantifying Vesicular Acetylcholine Transporter In Vivo in Humans using [^18^ F]VAT

Reliable quantification of cholinergic nuclei and terminal fields is possible using PET tracers that target the vesicular acetylcholine transporter (VAChT). Two such probes have recently been developed: [^18^F]FEOBV^49^ and [^18^F]VAT^25^. FEOBV has been used in rodents^50,51^, nonhuman primates^51^, and healthy human volunteers^49^, ^41,52–54^. Quantifying FEOBV is limited by slow kinetics that necessitate using either long scan times or short semi-quantitative static scans that rely on estimates of non-equilibrium tissue ratios including standardized uptake value ratios (SUVR). Tu et al generated [^18^F]VAT by modifying FEOBV’s structure. Early studies in rodents^25^ and non-human primates^26,27^ demonstrate that VAT has the faster kinetics necessary for fully quantitative measurement of VAChT throughout the brain. Based on these findings, we chose to utilize [^18^F]VAT for PET imaging of cholinergic terminal field integrity in an elderly population of humans in vivo. Using metabolite-corrected arterial plasma [^18^F]VAT concentration as input, we estimated VAChT distribution volume in key regions of interest throughout the brain. We were able to demonstrate differential terminal field loss in entorhinal cortex vs. hippocampus and somatosensory cortex in subjects who showed cognitive deficits on both the MOCA and the PennCNB SVOLT tests. Notably, these individuals were not recruited based on an existing clinical diagnosis but were part of a healthy community-based cohort. These results indicate that there are quantifiable losses in cholinergic terminal integrity in the EC in healthy individuals that correlate with reduced performance on cognitive tasks.

Previous studies have evaluated cortical FEOBV SUVR in participants recruited with a diagnosis of MCI^53^ or AD^52^. These studies have identified global deficits in cortical cholinergic innervation in established cognitive impairment^53^. We evaluated a population without clinical diagnosis of cognitive impairment, albeit with subjective memory concerns. Our studies extend upon existing findings to assess the integrity of cholinergic circuitry early in cognitive impairment and probe the cholinergic mechanisms underlying impaired EC function.

We focused our a priori analyses in both humans and mice on the circuit between the basal forebrain and EC based on reported early issues with EC-related functions with age ^17,19^. Our results are consistent with the hypothesis that loss of EC cholinergic terminal density might underlie some of the earliest phases of cognitive decline associated with age. We propose that the EC cholinergic deficits likely precede more robust global cortical deficits. Our ongoing longitudinal studies with larger participant cohorts and improved PET resolution are investigating the importance of LEC-specific cholinergic terminal integrity as the earliest predictive factor for future cognitive impairment.

## Conclusions

In a series of translational experiments, we present data supporting loss of cholinergic innervation in the LEC and loss of function of LEC-projecting cholinergic neurons as an early step in the aging trajectory and intimately related to early cognitive decline. Furthermore, we reveal an important role for ACh in normal EC functional engagement and object location memory. Our data suggests EC VAChT availability may be a sensitive biomarker for early detection and potential intervention in age-related cognitive decline. If the goal of the field is to find biomarkers for early intervention, it seems we are still looking too late! At this early stage of cognitive impairment, the BFCN◊EC circuit is already affected. Using these valuable in vivo imaging tools, supported by parallel preclinical assessment, studies that evaluate the onset of the cholinergic lesion and understand the predictive capability of VAChT density in diagnosing future cognitive impairment are needed. In addition, we find that while some cholinergic circuits are dramatically affected at this point, some remain intact. This concept falls in line with a growing body of literature supporting the heterogeneity of different central cholinergic populations^1,55,56^. Understanding what confers differential resilience and vulnerability to these populations may be key in maintaining cognition long-term.

## Methods

### Subjects

#### Human Subjects:

This study was approved by the Institutional Review Board at Stony Brook University. All participants provided written informed consent. Recruitment took place between September 2017 and June 2019.

Participants for the human study were recruited from the local community based on the following inclusion criteria: 1) Age between 50–85 years; 2) Capacity to consent; 3) Under 275 lbs (based on scanner bed weight limits). Exclusion criteria included: 1) Significant physical illness; 2) History of DSM-V disorder excluding substance use or dependence; 3) Current substance use disorder; IV drug use in the past 5 years, MDMA use more than 10 times in 5 years; 4) Nicotine use, including tobacco, e-cigarettes, nicotine patch, nicotine gum, or lozenges in the past year; 5) Women who are premenopausal or not surgically sterile; 6) Unable to stop drugs or medication that affect cognition including those that affect the cholinergic system; 7) Current, past, or anticipated exposure to radiation including being badged for work, participation in nuclear medicine procedures, or recent exposure to multiple radiographic images that would exceed yearly exposure limits; 8) Any MRI contraindications including claustrophobia, metal implants, pacemakers, ICDs; 9) Blood donation within 8 weeks of the start of the study; 10) History of head trauma with prolonged loss of consciousness or any neurological condition; 11) Medicinal patch that could not be removed; 12) Anticoagulant or anti-platelet treatment other than aspirin. Fourteen participants were recruited into the study and signed informed consent forms. Subjects were divided into two groups based on performance on the Montreal Cognitive Assessment (administered during the intake session): Older Adults (OA) presented with a MoCA score of 26 or greater; Impaired Older Adults (OA-I) presented with a MoCA score of less than 26. One OA participant did not complete the PET study procedures and was excluded from PET analyses. One OA-I participant did not complete PET and MRI study procedures and was excluded from PET and MRI analyses. These participants were included in the reporting of cognitive testing measures.

#### Animal Use:

Male and female mice between the ages of 1.5 and 6 months were used for the reported experiments. The following strains were used in the generation of this data: C57BL/6J (Jax stock number 000664), 5XFAD (Jax stock number 34840), NOS2−/− (Jax stock number 002609), Chat-*ires*-CreΔneo (Jax stock number 031661), and Chat-tau:eGFP (Sukumar Vijayarhagavan^34^). Aß^+^Tau^+^ animals were the offspring of crosses between 5XFAD^+^/NOS2^−/−^ X /NOS2^−/−^ : 5XFAD^+^ and 5XFAD^−^ mice were used as Aß^+^Tau^+^ and littermate controls. All mice were maintained on a C57 background. Animals were housed in a 12 hour light/dark cycle environment that was both temperature and humidity controlled. Animals had free access to food and water. Mice were either pair or group housed when possible. No single housed mice were used for behavioral experiments. All animal care and experimental procedures were approved by the Institutional Animal Care and Use Committees of the SUNY Research Foundation at Stony Brook University (1618) and the NINDS IRP (1490 and 1531).

### Procedures & Cognitive Testing

#### Clinical Procedures:

All human participants underwent a structured clinical interview for DSM-V (SCID) by a graduate level rater for evaluation of DSM-V disorders or diagnoses. All participants underwent a series of cognitive testing including a Mini Mental State Examination (MMSE), Montreal Cognitive Assessment (MoCA), and Penn Computerized Neurocognitive Battery (PennCNB). Analysis focused on the spatial visual object learning task (SVOLT task) within the PennCNB. Performance score was calculated by evaluating the number of correct responses for each participant within each test session consisting of 30 trials (score out of 30).

#### Mouse Stereotaxic Surgery:

3-month old Chat-*ires*-Cre mice or Chat-tau:eGFP mice were anesthetized and stereotaxically injected (Kopf) bilaterally (200nL per site) in the lateral EC (−4.0mm A/P, ±4.5mm D/V, −3.5mm M/L, empirically determined using landmarks). Viruses: CAV2-DIOhM4Di. mCherry was obtained from Dr. EJ Kremer (Institut de Génétique Moléculaire de Montpellier, France) and AAV9-PCaMKIIa-GCaMP6f.WPRE.SV40 (obtained from U.Penn Vector Core) to visualize the injection site.

Retrograde tracing experiments were conducted via injection with Fast Blue (3% solution in sterile water w/v, Polysciences Inc). 80nL of 3% Fast Blue was injected into the lateral entorhinal cortex of WT animals bilaterally. Mice were euthanized 4 days after injection.

#### Mouse Displaced Object Recognition (DOR) Task:

All mouse behavioral experiments were conducted in a dark room with overhead red-light lamps. The behavioral arenas used were 30cm × 30cm rectangular cages covered with a transparent lid. One arena was used for staging while the other was used for testing. Mice were transported to and from the home-cage using a transparent, plastic platform. The displaced object recognition task was conducted over five days. Each arena was wiped down with 70% ethanol or Clidox prior to the sessions to ensure the same scent was associated with each session and to eliminate the odor from any previous animal. Habituation took place on days one through four. Mice were habituated to both the staging and testing arenas as well as transport to and from the home cage. During habituation sessions, mice were given 5 minutes in each chamber before being returned to the home cage. The staging arena was empty (open arena), whereas the testing arena consisted of three objects spaced throughout the chamber. On day five, one of the objects was moved to a different location within the testing arena. Test sessions were 5 minutes. The displaced object was counterbalanced to ensure there was no preference to one side of the arena. Behavior data was recorded with a Logitech webcam and subsequently analyzed using Ethovision Analysis.

For DREADD experiments, mice were administered 0.1 mg/kg Clozapine^57^ (Sigma Aldrich) interperitoneally 10 minutes prior to the test session.

#### Mouse In Vivo Electrophysiology:

Mice were anesthetized with isoflurane and placed on a heated surgical stereotaxic stage (Kopf instruments). Craniotomy was performed over the left entorhinal cortex and a 5 MOhm parylene-C insulated tungsten electrode (AM systems, Sequim, WA) was placed into the left lateral EC. Recordings were pre-amplified using a head-stage from an A-M Systems amplifier. Data was acquired at a sampling rate of 40kHz, filtered between 100–1000 Hz by an A-M Systems amplifier and a Humbug Noise Eliminator (A-M Systems), and input to a 1401 data acquisition board (Cambridge Electronic Design). All data were collected using the Spike 2 software (Cambridge Electronic Design).

### PET/MRI Scanning Procedures

Radiosynthesis of [^18^F]VAT for in vivo Human Studies: [^18^F]VAT was prepared in a one-step synthesis using a GE Tracerlab FXN pro synthesis module. Briefly, [^18^F]fluoride was reacted with 2-((7-(4-(4-fluorobenzoyl)piperidin-1-yl)-6-hydroxy-5,6,7,8-tetrahydronaphthalen-1-yl)oxy)ethyl 4-methylbenzenesulfonate in acetonitrile. Unreacted [^18^F]fluoride was removed by passing the reaction mixture over an alumina SPE cartridge and [^18^F]VAT was separated from the other compounds in the reaction mixture using preparative high performance liquid chromatography (HPLC) using a C18 column (Phenomenex, 250 × 10 mm, LunaR 10ƒÊm C18(2) 100 A) and a mobile phase of 0.1 M ammonium formate in 38 % acetonitrile at a flow rate of 4 mL/min. The peak corresponding to [^18^F]VAT was collected and further purified by dilution with water and absorption onto a C18 solid phase extraction cartridge (SPE). The SPE was washed with water to remove traces of acetonitrile and buffer. The purified [^18^F]-VAT was then eluted with absolute ethanol and diluted with sterile 0.9% saline. The manufacturing process typically takes one hour, and the typical decay corrected chemical yield was 20–30%. Specific activities at the end of synthesis were 4.04 +/−1.55 Ci/μmole and the radiochemical purity was >99%.

Human Positron Emission Tomography Protocol: A venous catheter was placed for injection of the radioisotope. An arterial catheter was placed in the opposite arm for collection of arterial blood samples for the duration of the scan. PET and MRI data were acquired on a simultaneous PET/MRI scanner, Siemens Biograph mMR (software version VB20P). Up to 5 mCi of [^18^F]VAT was administered intravenously as a bolus over 30 seconds. Emission data was collected in three-dimensional mode for 150 mins post injection for 38 frames of increasing duration: 6 frames at 20sec, 6 frames at 1min, 6 frames at 2 min, 14 frames at 5 min, 6 frames at 10 min. PET data were reconstructed with the template-based Boston attenuation correction^58^ , scatter correction and randoms correction The intrinsic resolution of the scanner at the center of the field of view was 3 mm full width at half maximum.

#### Human MRI Imaging Protocol:

T1-weighted structural MRI images were acquired using a magnetization-prepared rapid gradient-echo (MP RAGE) sampling sequence. All images were acquired simultaneously during the PET acquisition with a 20-channel head coil and the following parameters: TR/TE/TI = 2300/2.98/900 ms, Flip Angle = 9°, IPAT (integrated parallel acquisition technique) = 2, and voxel resolution: 0.87 × 0.87 × 0.87 mm^3^.

#### Diffusion MRI images were acquired using a multi-band EPI sequence with the following parameters:

TR=6300ms, TE=121.4ms, flip angle=78 degrees, refocus flip angle=160 degrees, fat saturation, FOV=224×216×128mm, base resolution=112, bandwidth=992 Hz/Px, voxel size=2 × 2 × 2 mm^3^, multi-band acceleration factor=2, 4 shells of diffusion vectors with b values = 1000, 2000, 3000, 4000 with 64, 64, 32, 32 diffusion directions respectively, and an acquisition time of 18:16 minutes.

#### PET Metabolite & Free Fraction Analysis:

Arterial samples were collected with an automated sampling system (Swiss-Trace) continuously for the first 6 minutes and manually thereafter. Eleven arterial blood samples were obtained at 2, 6, 12, 18, 25, 30, 40, 50, 60, 120, and 150 min during each scan for measurement of the arterial plasma activity over time.

Seven plasma samples were manually obtained at 2, 6, 12, 18, 30, 60, and 120 min during each scan for the determination of intact and metabolized [^18F^]VAT. Samples of whole blood and plasma were weighed and counted in a gamma counter (Perkin Elmer Wizard, 2480) to determine the clearance rate. Plasma proteins were precipitated with acetonitrile and the supernatant analyzed by HPLC (LabLogic Posiram BGO coincidence detector) using a C18 column (Phenomenex, 250 × 4 mm, LunaR 10ƒÊm C18(2) 100 A) and an eluant of 0.1M ammonium formate in 50% acetonitrile, at a flow rate of 1 mL/min. The free fraction was determined by spiking the participant’s free plasma, pooled plasma and saline with [^18^F]VAT and incubating the mixture for 5 minutes. These samples were then centrifuged (Millipore, UltracelR PL) and samples of the original samples and the filtrates weighed and counted in a gamma counter (Perkin Elmer Wizard 2480).

The seven unmetabolized parent fraction levels were fit with a Hill Function and weighted equally. This fit was then used to correct the plasma radioactivity (multiplied by the plasma data), which was fit as a straight line to the peak, and the sum of three-exponentials after the peak. The fitted values were used as input to subsequent analyses.

### Analysis

#### Human PET/MRI Image Processing:

Imaging analysis was processed through our custom Brain Analysis Toolbox (BAT) consisting of a series of processing steps for both the MRI and the PET image. To begin, the last PET 30 frames are registered to the eighth frame using the Functional Magnetic Resonance Imaging of the Brain’s Linear Image Registration Tool (FLIRT; FMRIB Image Analysis Group, Oxford, UK) to correct for motion throughout the scan. Once the mean motion correction image is created, eight different registrations (between the mean PET image and the MRI) are considered using different weighting schemes as previously described^59^. Subject MRIs were preprocessed with SPM for segmentation (into grey matter, white matter, and CSF; Statistical Parametric Mapping, Wellcome Centre for Human Neuroimaging) and an automated skull-stripping algorithm (Atropos)^60^. After the optimal registration is chosen based on a mutual information metric and manual verification, the coregistration is applied to all PET frames and images are registered to the higher resolution MRI.

Three atlases were used in the quantification of regions reported in this manuscript. Entorhinal cortex (EC) regions (Figure 2/4) and corpus callosum (reported in the Extended data table) were defined using the Desikan-Kiliany atlas provided in FreeSurfer 5.3.0 (see below for FreeSurfer methods). Basal forebrain regions (CH1/2, CH3, and CH4p; Figures 3/**S2**) were defined as previously described^30^. The EC, CC and basal forebrain atlas regions were warped onto individual subject MRIs using Advanced Normalization Tools (ANTs)^61^. For all remaining regions reported (Figure 2/Extended data table) we generated probabilistic ROIs using our in-house probabilistic ROI atlas. In brief the atlas was generated as follows: 34 regions were manually hand-drawn onto 18 MRIs by experienced technicians guided by brain atlases^62,63^ and published reports^64,65^ as previously described^66^. Each template was registered to the target MRI using the Automatic Registration Toolbox (ART)^67^. Regional labels for each voxel were then determined by evaluating the percentage of the 18 brains that were labeled at that region. The probabilistic labels were used in the calculation of the regional time-activity curves (TACs). Final ROI placement was confirmed by visual inspection before proceeding.

#### Partial Volume Correction of Human PET Images:

Partial volume effects were corrected using the Iterative Yang technique^68,69^ from the PETPVC Toolbox^70^. Instead of calculating the regional mean values via the geometric transfer matrix, the values are estimated from the PET data itself. The Yang correction is applied, and the mean value estimates are recalculated, iteratively, 10 times. The partial volume corrected PET images were then fit for subsequent analyses.

#### [^18^F]VAT Distribution Volume Quantification:

To quantify tracer binding, time activity curves from partial volume corrected images along with the arterial input function were analyzed by using a standard 2-Tissue Compartment Model^71^. Subsequent analyses compared the primary outcome measure, distribution volume (V_T_)^71^. Outcomes were log-transformed to stabilize region-wise variance and to fulfill normality assumptions of subsequent model analyses. Hemispheres were plotted and considered individually.

#### FreeSurfer Processing of Human MRI Images:

Using MP-RAGE images, cortical thickness and volume were quantified for 34 left and 34 right hemisphere regions defined by the Desikan–Killiany atlas using FreeSurfer 5.3.0 (http://surfer.nmr.mgh.harvard.edu/). Freesurfer processing steps include skull-stripping, Talairach transformation, subcortical grey/white matter segmentation, intensity normalization, gray/white matter tessellation, topology correction, and intensity gradient based surface deformation to generate gray/white and gray/cerebrospinal fluid surface models. The resulting surface models were then inflated and registered to a spherical surface atlas, allowing parcellation of cortical regions of interest. The final outcomes generated were regional entorhinal cortical thickness and volume (Table 1) computed by averaging the white matter-to-pial surface distance at all vertices within the region (CT) or summing the total volume of the region. FreeSurfer EC ROIs were also used for PET analysis (see above).

#### Human Diffusion MRI Image Analysis:

All T1 and diffusion images were examined for common artifacts such as ghosting and ring-motion using a standardized examination procedure. Diffusion MRI images were loaded into DSI Studio and converted into ‘.src’ files after correction for eddy currents, motion, and phase distortion. Images were reconstructed using Q-space diffeomorphic reconstruction (QSDR) to the adult human template image at 2mm resolution with a 1.25 sampling length ratio for resolution of fiber tracts and fiber orientation. Regions of interest were defined using previously described basal forebrain subregion atlases^30^, and the FreeSurfer entorhinal cortex region. Automated, regional tractography was conducted by setting the MS/DB region as the seed and the entorhinal cortex as End. Diffusion metrics were exported from the visualized tracts for each participant and group metrics were calculated thereafter. The diffusion tensor metrics (FA, MD, AD, etc) were calculated using images with b values = 1000.

#### Mouse Tissue Processing:

Following PFA perfusion, mouse brains were post-fixed overnight at 4°C in 4% PFA (in 1XPBS) and were then transferred to a 30% sucrose solution (in 1XPBS). Brains were flash frozen in OCT Compound (Tissue Tek) and stored at −80°C until cryosectioning. 50 μm cryosections were collected into a solution of 1:1 PBS:glycerol and stored at −20 until immunostaining and/or imaging procedures.

#### Tissue Immunohistochemistry:

Sections were blocked overnight at 4°C in a PBS solution containing 2.0% TritonX-100 and 10% normal donkey serum and then incubated with primary antibody in a PBS-T solution (2% TritonX-100 and 10% normal donkey serum), overnight (24 h at 4°C). The next day, sections were rinsed in PBS-T and incubated in secondary antibody for 2 hr at room temperature in PBS-T solution (2% TritonX-100 and 10% normal donkey serum). Sections were mounted in Fluoromount-G (Southern Biotech). Primary Antibodies: Goat anti ChAT (Millipore, Cat# AB114P, 1:500), Rabbit Anti VAChT (Synaptic Systems, Cat# 139103, 1:500), Guinea-Pig Anti-cFos (Synaptic Systems, Cat# 226004, 1:500), Mouse-AT8 (Thermo Fisher, Cat# MN1020, 1:250), GP Anti ABeta (Synaptic Systems, Cat# 218308 , 1:500). Secondary antibodies: Donkey anti Goat IgG, Alexa Fluor 488 (Thermo Fisher, Cat# A32814, 1:750), Donkey anti Rabbit IgG, Alexa Fluor 647 (Thermo Fisher, Cat# A32795 1:750), Donkey anti Mouse IgG, Alexa Fluor 594 (Thermo Fisher, Cat# A32744, 1:750), Alexa-Fluor 647 AffiniPure Donkey Anti-Guinea Pig IgG (Jackson ImmunoResearch, Cat# 106-605-003, 1:750), Alexa-Fluor 488 AffiniPure Donkey Anti-Guinea Pig IgG (Jackson ImmunoResearch, Cat# 106-545-003, 1:750), Alexa-Fluor 594 AffiniPure Donkey Anti-Guinea Pig IgG (Jackson ImmunoResearch, Cat# 106-585-003, 1:750).

#### Cell Counts:

Tissue imaging was conducted on an Olympus wide-field slide-scanner microscope at 20X magnification (VS-120 and VS-200 systems, Z-step= 5 um), or an Olympus laser scanning confocal at 40X magnification (Z-step = 1um). Images were cropped using Olympus VSDesktop, and converted to ‘.ims’ files using the Imaris file converter. Using intensity-based thresholding and filtered by size, the ‘Spots’ feature was used to identify cFos+ cells within the 3D image. Using intensity based thresholding and filtered by size, the ‘Surfaces’ feature was used to identify ChAT+ cells within the 3D image. After identification of signal objects (cFos or ChAT+ cells), channels were masked to remove background. For colocalization analysis, masked channels were loaded into the colocalization tab within IMARIS and a new channel was created to include only colocalized pixels. This channel was then evaluated over the raw image and counted using the spots feature to identify colocalized cells.

#### Terminal Field Quantification:

To quantify terminal field density, images were collected on an Olympus laser scanning confocal at 40X magnification with Z acquisition over a 50um slice (Z-step = 1um). Images were loaded into ImageJ for further analysis. In ImageJ, images were split into individual channels, and the green channel was used for subsequent analyses. Images were then maximum-intensity-projected (MIP) to z-project across the optical sections. The images, in grey-scale, were then evaluated for grey-level value that served as threshold for signal vs. background (noise). Images were then cropped to equal ROIs using the crop to ROI function in ImageJ. Cropped images were saved as TIFs for subsequent processing. Next, cropped images were loaded into Matlab2018 and binarized (all background pixels were set to zero and all signal pixels were set to 255). Number of pixels in both background and signal groups was determined and the ratio of these was used as the final measure for terminal field density ratio.

#### In Vivo Electrophysiology Analysis:

Data was thresholded to remove breathing and heartrate signal. Extracellular recordings were sorted offline using the Offline Sorter (Plexon Inc., Dallas, TX). Features of the waveforms were extracted and individual units were demarcated by manually identifying clusters of waveforms in a 2-dimensional feature space of spike properties^72^. The quality of each sort was rated according to isolation distance between clusters. Only recordings of high sort quality, with less than 5% overlap with other clusters, were used for further analysis. Firing rate and inter-spike interval are examined between groups. Single unit recordings were then further analyzed using Matlab.

#### Ethovision XT15:

Behavior videos were loaded into Ethovision XT. An empty arena frame was gathered to define the analysis sections within the video. The arena area was subdivided into zones that included each of the three objects (Zones 1–3). The arena was additionally subdivided into quadrants (S1-S4). Keypoint tracking of the animal’s location was conducted throughout the video using nose, midpoint, and tail detection. Object exploration was defined as having a detection point within 2 cm of the object zone. Analysis profiles quantified time spent exploring Zone 1–3 separately, number of contacts with each zone, total object exploration, distance travelled. Data presented include time spent exploring the familiar vs. novel objects during the behavioral session.

#### Statistical Analysis:

Statistical analyses were done using GraphPad Prism (GraphPad Software Inc., V10, San Diego, CA, USA) or SPSS (IBM, V28 and V29). PET data was log-transformed for subsequent evaluation in linear mixed models. Normality of remaining data was assessed using Shapiro-Wilk and Smirnov-Kolmogorov tests. Data that failed either normality test were analyzed using non-parametric tests. Parametric tests used include generalized linear model (mixed models and ANOVA), and the Welch’s t-test (for unequal SDs). Non-parametric comparisons were conducted with a Mann-Whitney test. All boxplots display a median line with a box extending to the 25^th^ and 75^th^ percentile. Boxplot whiskers extend to min and max of the dataset.

## Figures and Tables

**Table 1:** Sample Characteristics

	Older Adults	Impaired, Older Adults	*p-value*

**Sample Metrics**			

**Total Participants**	**9**	**5**	--

**Age (years)**	**64.98** ± 7.7	**69.47** ± 8.8	0.28

**Sex (% female)**	**37.50%**	**100.00%**	0.01*

**Cognitive Measures**			

**MMSE (score out of 30)**	**28.78** ± 1.1	**25.2** ± 3.7	0.09

**MoCA (score out of 30)**	**27.56** ± 1.3	**21.4** ± 4.2	0.005*

**PennCNB: SVOLT (score out of 30 trials)**	**15.44** ± 1.6	**15.2** ± 1.8	0.8

**PennCNB SVOLT-D (score out of 30 trials)**	**15.11** ± 1.2	**10.8** ± 3.4	0.01*

**Imaging Metrics**			
**Injected Dose [^18^F]VAT (mCi)**	**3.84** ± 0.8	**3.77** ± 0.6	0.14

**Free Fraction (f_P_)**	**0.03** ± 0.003	**0.03** ± 0.01	0.44

**FreeSurfer Cortical Thickness: Entorhinal Cortex (mm)**	**3.15** ± 0.4	**3.09** ± 0.6	0.81

**FreeSurfer Cortical Volume: Entorhinal Cortex (mm^3^)**	**1475.67** ± 343.2	**1260.88**± 271.6	0.13

## Data Availability

The datasets generated and analyzed in this study are available from the corresponding authors on reasonable request.
